# Fault Tolerance in Protein Interaction Networks: Stable Bipartite Subgraphs and Redundant Pathways

**DOI:** 10.1371/journal.pone.0005364

**Published:** 2009-04-28

**Authors:** Arthur Brady, Kyle Maxwell, Noah Daniels, Lenore J. Cowen

**Affiliations:** 1 Center for Bioinformatics and Computational Biology, University of Maryland, College Park, Maryland, United States of America; 2 Department of Computer Science, Tufts University, Medford, Massachusetts, United States of America; Johns Hopkins University, United States of America

## Abstract

As increasing amounts of high-throughput data for the yeast interactome become available, more system-wide properties are uncovered. One interesting question concerns the fault tolerance of protein interaction networks: whether there exist alternative pathways that can perform some required function if a gene essential to the main mechanism is defective, absent or suppressed. A signature pattern for redundant pathways is the BPM (between-pathway model) motif, introduced by Kelley and Ideker. Past methods proposed to search the yeast interactome for BPM motifs have had several important limitations. First, they have been driven heuristically by local greedy searches, which can lead to the inclusion of extra genes that may not belong in the motif; second, they have been validated solely by functional coherence of the putative pathways using GO enrichment, making it difficult to evaluate putative BPMs in the absence of already known biological annotation. We introduce *stable bipartite subgraphs*, and show they form a clean and efficient way of generating meaningful BPMs which naturally discard extra genes included by local greedy methods. We show by GO enrichment measures that our BPM set outperforms previous work, covering more known complexes and functional pathways. Perhaps most importantly, since our BPMs are initially generated by examining the genetic-interaction network only, the location of edges in the protein-protein physical interaction network can then be used to statistically validate each candidate BPM, even with sparse GO annotation (or none at all). We uncover some interesting biological examples of previously unknown putative redundant pathways in such areas as vesicle-mediated transport and DNA repair.

## Introduction

It is estimated that only 18% of the yeast genome consists of essential genes, meaning that if the gene is deleted, the resulting strain is not viable on rich media [1]. Sometimes, the reason a given gene is not found to be essential is that the gene is not required for growth in rich media under laboratory conditions [2], [3]; for example, a gene which produces an enzyme used to metabolize one particular nutrient if other nutrients are available [4]. In other cases, however, genes are not essential because there exist other genes that can compensate for the missing gene. Three main mechanisms of compensation have been observed [2], [3], [5]. First, there can exist one or more paralogs of a nonessential gene which can substitute directly for it. The second mechanism involves the existence of redundant metabolic pathways or regulatory networks; this is called “robustness” by Wagner [6]. A third mechanism involving a more global and diffuse relation among multiple genes across many pathways has also been reported to occur [7]. There is only preliminary data on the relative importance of the three mechanisms – one study estimates that at least 25% of the gene deletions in yeast that have no phenotype involve the first mechanism of duplicate genes [8].

Recent years have seen a huge increase in the amount of genetic interaction data available from yeast double-mutants, where interactions between pairs of nonessential genes are characterized by the phenotypic effect of their simultaneous suppression or deletion. One of the simplest of such effects is a “synthetic-lethality” interaction: both genes are nonessential, but their simultaneous deletion destroys the viability of the yeast. A synthetic-lethality genetic interaction (GI) network is defined by representing genes/proteins as nodes, with an edge between two nodes if a synthetic-lethality interaction has been observed between the corresponding genes. An increasingly comprehensive protein-protein physical interaction (PI) network (defined as was the GI network, with nodes as genes/proteins and edges as pairwise interactions) is available for yeast, where physical interactions include direct binding between two genes' protein products, regulatory protein-DNA binding mechanisms, and the existence of enzymatic reactions between pairs of proteins linked by a common metabolite (excluding common metabolic cofactors like water and ATP [7]).

In a seminal paper, Kelley and Ideker [7] showed how the superimposition of the PI and GI networks could be used to search the yeast interactome for a simple network sub-architecture that they called a *between-pathway model* (BPM). The search for BPMs within the yeast interactome was studied further by Ulitsky and Shamir [9], and by Ma, Tarone, and Li [10] using different models. The BPM model treats GI and PI edges as fundamentally different. This is in contrast to the model used by Nabieva et al. [11] in their groundbreaking work using maximum flow methods to predict gene function: their method depends on GI edges being treated as simply one-type of high-confidence PI edges. In the search for fault-tolerance, in contrast, it is crucial that these two types of edges be treated separately: the fundamental insight of this paper comes from recognizing that we can view the PI edges as ordinary edges, and the GI edges as *2-vertex cuts* of the functional network. Thus algorithmic work related to the theory of maximum (and in our case maximal) cuts becomes highly relevant.

**Figure 1:**
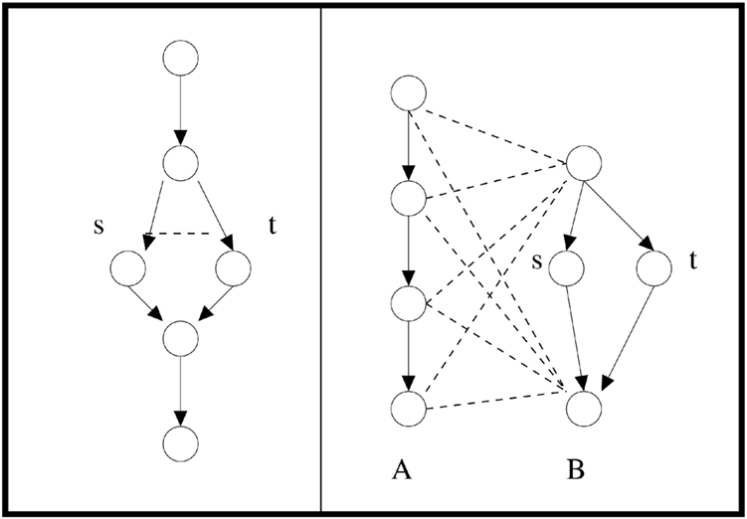
Redundant pathway example. A solid arrow denotes a physical interaction; a dashed line denotes a synthetic-lethality interaction. In the graph on the left, deleting either gene s or gene t will still allow for the successful traversal of the pathway. In the graph on the right, there are two alternate redundant pathways A and B. Note synthetic-lethality edges do not form a complete bipartite subgraph, because gene s and gene t supply lower-level redundancy. The st double mutant is synthetically lethal in the graph on the left, but not on the right, where it does not block alternate pathway A.

Specifically, a BPM is a graph-theoretic indicator that genetic fault tolerance may be present. Consider a model consisting of a pair of protein pathways where each pathway serves as a redundant backup for the other. *Within* each pathway, there will be many physical interactions between nodes (protein-protein binding, direct transcriptional regulation, etc.), reflecting each pathway's existence as a coherent functional unit. Synthetic-lethality interactions, on the other hand, will be few or nonexistent *within* each pathway, since the other pathway provides a failsafe mechanism for its partner. *Between* the two pathways, there will be more observed synthetic-lethality interactions: if corresponding components are deleted or suppressed in both pathways at once, the fault-tolerance of the system is defeated and the strain dies. A network motif corresponding to this situation, in which two groups of genes – each group found to be edge-dense within the PI network – are connected by many synthetic-lethality edges in the GI network, defines the BPM (see Figure 1).

Kelley and Ideker, and later Ulitsky and Shamir, identified possible examples of the BPM architecture for yeast. They found that many of their candidate BPMs were enriched over certain Gene Ontology (GO) categories of protein function [12], and correlated well with some biologically-known examples of pathway buffering. However, in both cases, a heuristic approach was used to extend small connected components of the PI network, searching for patterns in a combined network which superimposed both physical and genetic interactions.

In contrast, our new method initially takes into account only the graph-theoretic structure of the synthetic-lethality GI network to search for candidate BPMs; this allows the location and density of physical interactions in the PI network to be used afterward to validate the results. (We note that a recent paper, Ma et al. [10], also takes an approach based on GI edges only; but their strategy is to produce a very large set of possible candidate BPMs, many of which will be meaningless, which must then be filtered using GO annotation to discover a much smaller subset of meaningful pathways; we discuss this more below).

The subgraphs of the synthetic-lethality network which our method returns as putative examples of the BPM architecture we call *stable bipartite subgraphs*. They are defined as follows. Given any bipartition of all nodes of a network into sets *A* and *B*, we call such a partition *maximal* if the act of moving a single node from *A* to *B* or from *B* to *A does not increase* the number of edges crossing between *A* and *B*. These partitions are locally maximal; there can be many different maximal bipartitions of the same network. There exists an efficient, randomized, greedy algorithm [13] for sampling maximal bipartitions in any network, described below. (In contrast, finding the bipartition with the globally maximum number of edges crossing between *A* and *B* is NP-hard. [14], [15])

Given the results of *M* repeated runs of the randomized maximal bipartition detection algorithm on a network, we define the *stable bipartite subgraph of* any node *v* to be the bipartite subnetwork (with bipartition 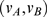) consisting of all nodes in the network (set 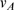) that appear in the *same* partition as *v* in at least 70% of sampled maximal bipartitions, and all nodes (set 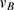) that appear in the *opposite* partition from *v* at least 70% of the time. The putative BPMs returned by our method consist of the stable bipartite subgraphs generated for each node *v*. Note that we obtain fewer stable bipartite subgraphs than genes, because some genes generate the same stable bipartite subgraph.

We show that BPMs obtained from stable bipartite subgraphs show significant functional enrichment over GO categories (using FuncAssociate [16], using an FDR multiple testing correction). Using a network containing the same set of GI and PI edges as that explored by Kelley and Ideker (i.e. the edges known in 2005), we find 602 BPMs covering 1,526 SL edges with 53.4% of the 602×2 = 1204 putative functional pathways exhibiting GO enrichment (for some functional category of depth ≥3); Kelley and Ideker reported 360 BPMs covering 687 SL edges with 34.9% of their 360×2 = 720 putative pathways exhibiting GO enrichment. Using a more recent network of GI and PI edges from the BioGRID [17] (as of October 2007), we find 50.6% of 3020 pathways exhibiting GO enrichment, as compared to Ulitsky and Shamir, who find less than 36% of their smaller number of pathways enriched, on a similar but slightly older network (see Table 1). Furthermore, coverage of known complexes by our BPMs is substantially increased over those of Ulitsky and Shamir (79.8% of the complexes annotated in SGD GO-slim [18] for our BPMs, versus 46.3% for theirs).

**Table 1:** Coverage of the interactome by BPMs.

	Network	BPMs found	SL edges covered	Fraction Enriched Pathways
Kelley/Ideker	*G*	360	687	251/720 (34.9%)
**Our results**	*G*	**602**	**1,526**	643/1204 (**53.4%**)
Ulitsky/Shamir *A*	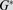	140	<3,765	100/280 (35.7%)
Ulitsky/Shamir *B*	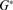	270	<3,765	177/540 (32.8%)
**Our results**	*G′*	**1,510**	**4,949**	1528/3020 (**50.6%**)

*G* is the exact same set of interactions that was known to Kelley and Ideker in 2005; *G′* is the more recent BioGRID network; G^*^ is the slightly older network used by Ulitsky and Shamir. Ulitsky and Shamir's results *A* and *B* come from their supplementary Table S1, where *A* was generated using their “dense pathways” method and *B* using their “connected pathways” method. Ulitsky and Shamir treat synthetic-lethality and synthetic-sick interactions as equivalent; they cover 3,765 interactions in the combined set. Each BPM contributes two pathways to the enrichment calculations; we consider a pathway GO-enriched if the GO term has a depth of at least three in the hierarchy and *p*≤0.01 using FuncAssociate with an FDR multiple-testing correction. Both Kelley and Ideker as well as Ulitsky and Shamir use *p*≤0.05 with a hypergeometric test.

Since the PI edges are not considered by our BPM construction method, we can go on to measure the propensity of physical protein-protein interactions to occur *within* rather than *between* putative pathways. Using this measure, we obtain high-confidence pathways that are *not* currently represented in known functional annotation; thus we can make new biological functional and fault-tolerant predictions. As further statistical validation, we find that the BPM motifs which we predict from the smaller Kelley and Ideker interaction network are consistently carried forward on the larger BioGRID network. That is, newly-discovered synthetic-lethality relationships and protein-protein interactions (which appear in the BioGRID data after 2005) tend to appear where we would expect from the structure of the BPMs generated from the older network.

All of our candidate BPMs, along with enrichment results and individual constituent gene annotations, are publically available at http://bcb.cs.tufts.edu/yeast.bpm/. When we refer to a BPM by number in this paper, the number refers to the ID associated with that BPM on this website.

## Results

Two datasets describing the yeast interactome were studied: the first contained the interaction data used by Kelley and Ideker (KI) in [7], whose synthetic-lethality (SL) network we denote by *G*. The second includes the first as well as an updated collection of all additional SL and protein-protein interactions published in the October 1, 2007 release of the BioGRID database [17], which we denote by *G′*. Both datasets were filtered to exclude essential genes, as well as all genes not found to participate in any synthetic-lethality relationships. Thus filtered, *G* contained 682 gene/protein-product nodes with 1,858 synthetic-lethality interactions, and *G′* contained 1,678 genes with 6,818 SL edges. This data represents only a fraction of the total estimated number of SL interactions in the yeast interactome, because most gene pairs have not yet been tested (we address complications arising from the incompleteness of the known interactome below).

We computed the stable bipartite subgraph of each gene in *G* and *G′*; note that for some genes participating in the same BPM, their stable bipartite subgraphs will be identical, so fewer unique BPMs were generated than the number of genes in each network.

### Biological validation (GO enrichment results)

The number of different BPM subgraphs we found using this method, the total count of distinct SL edges involved in these BPMs, and the number of pathways found to be enriched for at least one GO category of depth 3 or more, is reported in Table 1, for the network *G* (identical to Kelley and Ideker's network) and for the more up-to-date network *G′*. Additionally, Ulitsky and Shamir report 46.3% coverage of the complexes annotated in SGD GO-slim [18]; our coverage of the same database was 79.8%. In both cases, we find many more BPMs on the comparable networks than do the previous studies. This is not surprising, because Kelley and Ideker, as well as Ulitsky and Shamir, include physical protein-protein interaction data in their search for BPMs, so one might expect they would find a smaller set of BPMs. It might be expected that a larger proportion of their BPMs would be enriched, since their BPMs are supported by both genetic and physical interaction data, whereas ours are based solely on genetic interaction data. In fact, Ma et al. [10], using another method that employed only SL edges to construct BPMs (as we do), found exactly this: that they generated more BPMs, but a smaller fraction were GO-enriched. Surprisingly, a *larger* proportion of the BPMs put forth by our method are enriched as compared with previous work: over 53% of our BPM pathways were enriched in *G*, and over 50% in *G′*, whereas the previous methods which used both networks to generate BPMs never exceeded 36%. We attribute this improvement to the power of the stable bipartite subgraph algorithm to automatically prune unrelated genes which are more often included by localized greedy heuristics.

We did not place the results of Ma et al. [10] in the comparison table; because some of their BPM generation rules bias the pathway samples, and not enough of their pathway data is available for us to generate valid comparison statistics. In particular, using a local greedy approach similar to Kelley and Ideker, but limited to the GI network, Ma et al. report 2,590 generated BPMs, but those BPMs were not made available; instead a subset of 89 BPMs from this set was published that satisfied the following criteria: 1) each pathway contains at least 4 genes, 2) both pathways are enriched for the same GO annotation, and 3) at least 30% of the genes in each pathway have GO annotations that match the annotation that is enriched for both pathways. Presumably, if these enrichment heuristics were relaxed, the number of enriched BPMs would increase from the 89 they report, but since the initial set of 2,590 BPMs was not published, it was not possible to determine by how much. Ma et al. do report that a smaller fraction of their pathways are enriched than those of Kelley and Ideker and Ulitsky and Shamir; in their discussion, they attribute this to their use of GI edges only. Our method used only GI edges and produced a *higher* percentage of enriched BPMs than the methods that use PI edges as well, so we suggest a different conclusion than Ma et al. concerning the amount of information present in the GI network by itself.

610 of the BPMs we found in *G′* had both pathways enriched; of these, all but 71 had at least one functional-enrichment term common to both pathways. A partial list appears in Table 2. We found 71 BPMs in *G′* for which both pathways were enriched for at least one GO term, but where no enriched GO terms were found which were *common* to both partitions. These might represent *interdependent* but not *redundant* pathways, or else might represent genuinely redundant pathways which have not yet been sufficiently annotated. A partial list of these appears in Table 3. In both tables, “Coverage” columns indicate how many genes – out of all genes in the background set matching the listed GO term – were found in each pathway. BPM number refers to the IDs given in the list of BPMs on our website (see above). We also found 308 BPMs where only one of two partitions exhibited GO enrichment of sufficient specificity.

**Table 2:** We considered the set of BPMs in *G′* for which both partitions were enriched for *the same* GO term.

BPM	Common GO term	Coverage
18: **UPC2 ECM22** MOT3 **ERG24**	sterol metabolic process	(3/12)
**ERG28** HAP1 **ERG6 ERG2**		(3/12)
19: **PHB1 PHB2 MDM31 MDM32**	mitochondrial envelope	(4/89)
**MDM10 MDM34 MMM1 ATP10 PSD1 ATP23 MDM12**		(7/89)
144: **DRS2 SNC1 APM3 LAA1 SLA2 SNC2**	vesicle-mediated transport	(6/164)
VPS3 **VPS45 CHC1 YAP1802 YAP1801 PEP12**		(5/164)
407: **MSTL1 STO1 CBC2**	spliceosome	(3/18)
**MUD1** NPL3 **NAM8 MUD2 SRV2 RTT106**		(3/18)
838: **DIE2 OST6 ALG6 ALG8 ALG5**	transferase act. - glycosyl grps	(5/36)
**PMT2 PMT1** TPS2 RP041 **OST5 OST3**		(4/36)

This table presents 5 of the top (nonredundant) matches, each with enrichment *p*-value<.0001 for the best-scoring GO term common to both partitions. Bolded genes are those annotated with the listed term.

**Table 3:** Top-scoring BPMs from among those which had both pathways enriched for *some* GO function, but whose GO matches were different across the two partitions.

BPM ID	Partition	Best-scoring GO term	p-value	Coverage
213	1	double-strand break repair via homologous recomb.	<0.001	(7/16)
	2	carboxy-terminal domain protein kinase complex	<0.001	(3/3)
324	1	actin filament depolymerization	0.002	(3/3)
	2	dynactin complex	<0.001	(3/3)
465	1	response to DNA damage stimulus	<0.001	(17/120)
	2	Ctf18 RFC-like complex	<0.001	(3/3)
567	1	microtubule cytoskeleton	<0.001	(14/36)
	2	nuclear microtubule	<0.001	(3/3)
720	1	response to DNA damage stimulus	<0.001	(8/120)
	2	RecQ helicase-Topo III complex	<0.001	(3/3)
778	1	nuclear lumen	<0.001	(8/171)
	2	carboxy-terminal domain protein kinase complex	<0.001	(3/3)
1076	1	DNA metabolic process	0.005	(15/384)
	2	mitotic sister chromatid cohesion	<0.001	(5/12)
1270	1	protein depolymerization	0.002	(4/9)
	2	microtubule-based process	<0.001	(13/46)
1338	1	nucleoplasm part	<0.001	(14/127)
	2	proteasome complex (sensu Eukaryota)	0.002	(4/12)
1357	1	peroxisome organization and biogenesis	<0.001	(6/18)
	2	fungal-type cell wall	0.003	(3/31)

An example GO term for which each pathway was enriched is provided, along with that term's associated *p*-value and coverage fraction.

### Mathematical validation (probabilistic results)

The first mathematical validation involves examining the physical protein-protein interaction (PI) network; if our BPMs represent real redundancy in function, PI edges should be biased to occur *within* each partition as opposed to *between* partitions. We measure, for each BPM, how much more biased the observed PI edges (between all pairs of gene/protein nodes in the BPM) are to remain within a single partition than would be expected by chance (see the Methods section for computational details). Of the BPMs we found (which were all generated using only synthetic-lethality interaction edges), the top 10 most strongly validated by the location of known PI edges (and their associated *p*-values) appear in Table 4.

**Table 4:** Ten of the top (non-redundant) dually-enriched BPMs, ranked according to the improbability of observed protein-protein interaction distributions appearing by chance.

BPM ID	Enrichment results	*p*
331	COMPASS,Rpd3L,SWR1 complexes; histone modific./chromatin remodel.	<1×10^−20^
465	Mre11 complex; DNA damage response	8.0×10^−7^
785	cytoskeleton; dynein complex; microtubule	7.3×10^−6^
944	Golgi apparatus part	1.7×10^−3^
201	CORVET, GARP, HOPS complexes; Golgi to vacuole transport	2.7×10^−3^
1043	ER to Golgi vesicle-mediated transport; regulation of pH	7.0×10^−3^
160	GET complex; intra-Golgi vesicle-mediated transport; secretion	0.010
1004	Mdm10/Mdm12/Mmm1 complex; mitochondrial envelope	0.012
1083	RecQ helicase-Topo III complex; recombination	0.012
778	DNA packaging; chromatin assembly complex	0.015

“Enrichment results” contains a brief summary of enriched GO terms for each BPM. “*p*” is the probability of seeing, by chance, the observed bias of PI edges to remain *within* one pathway rather than cross *between* the two pathways of the BPM.

The second statistical validation we applied to our approach was to check the consistency of the BPMs we generated using the Kelley and Ideker network *G* in the context of those generated from the more recent BioGRID dataset *G′*. Synthetic-lethality interactions in the newer BioGRID dataset are (except for a small number of false positives weeded out since 2005) a superset of the older data. If our BPMs are biologically meaningful, then, SL interactions reported since the Kelley and Ideker network was constructed should tend to appear between genes in *different* partitions of the BPMs generated from the older network. We therefore estimated the bias of the distribution of all such newly-reported SL interactions in favor of appearing *between* rather than *within* pathways (see the Methods section for computational details). Across the set of 175 BPMs from *G* which contained at least 20 *new* SL edges, the average probability that the observed between-pathway bias would occur by chance was 0.017. Since these new edges were not used to construct candidate BPMs in *G*, their distribution bias provides parallel independent support to the hypothesis that stable bipartite subgraphs do indeed correspond to biologically meaningful motifs.

### Example BPMs

**Figure 2:**
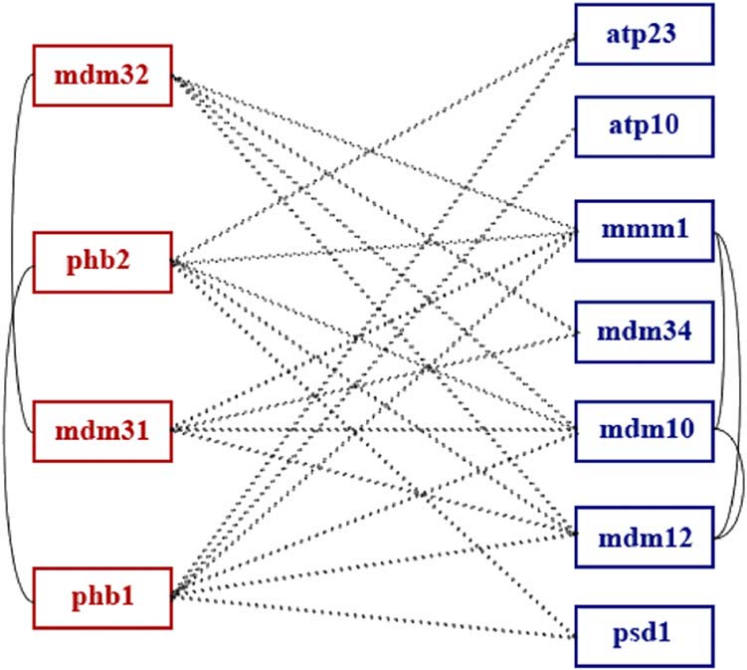
BPM 19 in *G′*. Synthetic-lethality interactions appear as dashed lines. Known protein-protein interactions appear as solid lines.

Figure 2 shows one example BPM from Table 2 in more detail. The first partition was enriched for both GO:0005743 (mitochondrial inner membrane) [p<0.001 (4/49)] and GO:0005740 (mitochondrial envelope) [p = 0.002 (4/89)], with all four genes in that partition annotated with both terms. The second partition was enriched for GO:0005740 (mitochondrial envelope) [p<0.001 (7/89)] (with all seven genes annotated with this GO term), as well as for GO:0005741 (mitochondrial outer membrane) [p = 0.001 (4/29)] (with four of the seven genes in this partition thus annotated).

Dimmer et al. [19] showed that deletion of *MDM31* or *MDM32* resulted in a very similar phenotype as deletion of *MDM10/MDM12/MMM1*, namely large, rounded mitochondria with profoundly reduced motility. On the other hand, deletion of *PHB1* or *PHB2* (the tumor suppression protein prohibitin and its homolog) displayed no detectable phenotype, but was found to be synthetically lethal when any of the genes *MDM12*, *MDM10* or *MMM1* on the right side of the partition were mutated [20]. The remaining two genes, 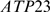 and 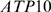, both associated with the mitochondrial envelope, are believed to possess overlapping functions with respect to ATPase biogenesis [21].

**Figure 3:**
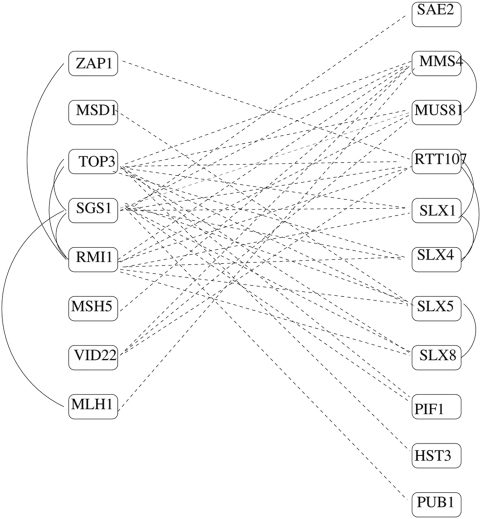
BPM 720 in *G′*. Synthetic-lethality interactions appear as dashed lines. Known protein-protein interactions appear as solid lines.

Figure 3 shows an example BPM from Table 3 in more detail; three genes on the left (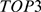, *SGS1*, *RMI1*) are known to make up the RecQ helicase-Topo III complex (G0: 0031422), while on the right, overlapping sets of genes are annotated as being involved in GO:0006974 (response to DNA damage stimulus) and GO:0006310 (DNA recombination). *SGS1* is the yeast homolog of *BLM*, responsible for the cancer-prone Bloom's syndrome in humans [22], [23], whose signature is cells with unregulated crossing-over. It is known to prevent aberrant crossing-over during meiosis by suppressing formation of joint molecules comprising three and four interconnected duplexes [24]. Hollingsworth and Brill [25] studied the endonuclease *MUS81-MMS4*, and showed that this two-protein complex also has a role in generating crossovers. In fact, they postulate that there are two *independent* mechanisms for resolving recombination intermediates, including holiday junctions, during meiosis: one involving *MUS81-MMS4*, and one involving the RecQ helicase-Topo III complex. They note that budding yeast appears to have the extra pathway as a failover, but that some other organisms appear to have evolved to exclusively use only one mechanism or the other. Our BPM appears to support this theory, while segregating additional genes, some already known to be involved in DNA repair, into association with one mechanism or the other. A literature search finds additional support: Wagner et al. [26] show that *PIF1* has a direct role in the prevention or repair of *SGS1*-induced DNA damage that accumulates in top3 mutants. Mullen et al. [27] propose that the *MMS4/SLX3*, *SLX5/8*, and *SLX1/4* gene pairs encode heterodimeric complexes and speculate that they are required to resolve recombination intermediates arising in response to DNA damage, during meiosis, in the absence of 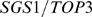.

### Ascertainment bias

While not addressed in the work of Kelley and Ideker or Ulitsky and Shamir, Fritz Roth [28] alerted us to an issue of possible *ascertainment bias*, based on in the available synthetic-lethality data, which needs to be addressed. In particular, many smaller-scale synthetic-lethality experiments result in data with an artificially bipartite structure. That is, they test a set of query genes against a set of genes on an array, and query genes were only tested against array genes and not against each other. A complete graph could therefore artificially appear in the data as bipartite, based on which subset of all possible gene pairs was tested. We note that the strong enrichment results obtained both in this study and in previous work go some way toward implying that we are not just rediscovering bipartite structure in the network left by ascertainment structure; support for the relevance of our BPMs is also deepened by our validation results concerning the observed within-versus-between distribution bias of protein-protein interactions, as well as validation based on the biased distribution of newly-tested synthetic-lethality interactions, appearing where we would predict them to appear as more experimental data is generated. Even so, we wished to quantify the extent to which ascertainment bias could be affecting our results.

We ordered the various experiments that produced synthetic-lethality data in the BioGRID dataset by volume, according to the number of synthetic-lethality interactions each contributed. Thus ordered, the top 25 experiments taken together contributed 72% of all synthetic-lethality interactions in the database. For these experiments, we went through each of the associated papers and uncovered exactly which pairs of genes were tested for synthetic-lethality relationships. In this way, instead of having two labels for SL interactions (“known to be synthetic-lethal” vs. “known not to be synthetic-lethal or never tested”), we now had three possible labels (“known to be synthetic-lethal”, “known not to be synthetic-lethal” and “never tested”). Intuitively, a BPM could be an artifact of ascertainment bias if it turned out that all or nearly all pairs of genes tested for synthetic lethality turned out to lie *between* the two pathways, with few or no tests having been performed between pairs of genes that lie *within* the same pathway.

As an example, consider the “worst” BPM we are able to find in our set, BPM 622. There are four genes in one pathway (call it pathway 1) that were tested by hand across several very small-scale experiments (not in the top 25 by volume): *ECM1*, *PHB1*, *PHB2*, and 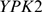. In pathway 1 is also *HSP92*, which was a query gene in a very high-throughput experiment. In pathway 2, we find 5 genes that were also tested in the small-scale experiments (*MDM10*, *RPL2A*, 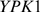, 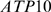, and *MDM12*), but there are an additional 128 genes which were array genes in the same high-throughput experiment in which *HSP92* was a query gene. Further examination shows that *none of the pairs of these 128 array genes were ever tested against each other*; thus, most of the genes in this 133-gene partition are likely to be present simply as an artifact of ascertainment bias.

At the other extreme, we are more confident of those BPMs where, for example, many pairs of genes *within* each pathway were tested for synthetic lethality. Considering only the top 25 experiments (so this is an underestimate), we find that at least 391 out of 610 dually-enriched BPMs had at least 10 pairs of genes tested in pathway 1, together with at least 10 pairs of genes tested in pathway 2.

Denote the numbers of pairs of genes *known to have been tested for synthetic lethality* within pathway 1, between pathways, and within pathway 2 by A, B and C, respectively. Suppose there were *M* total synthetic-lethality edges observed within the BPM as a whole, and suppose 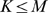 of these appeared between the two pathways. We compute the probability of observing, by chance, 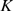 or more edges between the two pathways, when *M* edges are randomly assigned to the slots created by known tested pairs, given by
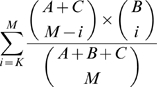



Table 5 lists the top 25 of our dually-enriched BPMs, ordered by this statistic. (We stress here that this statistic is *not* equal to the probability of observing one of our BPMs independent of ascertainment bias, because our BPM generation process will bias for edges going across; i.e. regardless of underlying structure, the placement of SL edges is not uniform, but biased by our algorithm to produce partitions where edges appear between pathways. Nonetheless, pathways which have a low value according to this *p* will have the desired quality that many edges within each pathway were, in fact, tested for synthetic lethality, thus we can still rank our confidence in the BPMs based on this *p*).

**Table 5:** The top 25 dually-enriched BPMs with respect to synthetic-lethality edge distribution.

BPM ID	In pathway 1	Between pathways	In pathway 2	*p*
1336	8/2743	340/609	9/59	1.44×10^−271^
1322	8/3696	317/889	4/45	1.93×10^−230^
515	13/5203	390/1528	21/117	3.82×10^−226^
1222	8/4551	354/1254	17/102	2.56×10^−221^
247	4/2108	207/298	0/15	4.85×10^−220^
984	6/2576	225/372	5/21	5.16×10^−220^
431	16/5049	347/1241	22/93	1.34×10^−216^
624	23/54	265/598	9/3600	2.23×10^−211^
723	5/2300	212/353	0/21	1.21×10^−209^
121	8/4285	362/1407	15/78	4.18×10^−205^
1426	17/3748	332/1066	23/153	6.95×10^−192^
642	3/2520	234/550	0/28	9.79×10^−192^
854	18/4432	358/1253	43/209	1.04×10^−191^
1133	19/4418	364/1282	43/195	3.33×10^−191^
672	12/5013	397/1830	34/232	5.16×10^−191^
151	18/4316	358/1238	43/195	5.70×10^−190^
588	29/4354	389/1394	40/206	2.06×10^−189^
926	11/3747	302/1013	15/99	1.53×10^−187^
379	20/4671	381/1435	51/216	3.79×10^−187^
1096	22/4205	382/1378	40/192	4.53×10^−187^
831	10/3604	279/919	7/75	1.59×10^−186^
789	17/4060	348/1214	33/169	1.60×10^−186^
978	17/4173	355/1227	43/195	5.67×10^−186^
1324	6/2284	225/425	11/35	7.69×10^−186^
692	26/4543	387/1422	51/216	6.70×10^−185^

“In pathway 1” represents the fraction of pairs of genes in the first pathway of the BPM which are *known to have been tested* for synthetic lethality, which actually exhibited an SL relationship. Likewise, “Between pathways” and “In pathway 2” list the observed number of SL interactions over the number of known tested pairs between the two pathways and within the second pathway, respectively. The last column lists the probability of observing by chance the bias of SL edges in the BPM in favor of appearing *between* rather than *within* pathways if edges were placed independently at random between all known tested pairs.

As an increasing fraction of all possible yeast double mutants are grown and tested for genetic interactions, the problem of ascertainment bias in the data with resolve on its own. In the meantime, in order to help the yeast genome researcher weed out those BPMs (like BPM 622, discussed above) which are likely to be artifacts of ascertainment bias, on our website at http://bcb.cs.tufts.edu/yeast.bpm/ we have annotated every gene in every BPM pathway with the names of the experiments from which it came, and whether it was a query or an array gene (the latter label provided only for the top 25 experiments by volume). Using this annotation, one can quickly flag BPMs in which query genes appear opposite array genes from a particular large-scale experiment. One can likewise easily identify cases where many pairs of non-edges were in fact tested for synthetic-lethality interactions, in which cases the likelihood of ascertainment bias is greatly reduced.

## Discussion

We have introduced the stable bipartite subgraph as a new means to generate redundant-pathway hypotheses in genetic and protein-protein interaction networks, and we have shown that this approach can generate subnetwork motifs (BPMs) that provide substantially more coverage than earlier approaches, with confident functional-enrichment results.

For the majority of our BPMs, we have evidence (in the form of either high-confidence enrichment results or well-characterized protein-protein interactions) that we are describing genuine redundant pathways. As for the rest, we examine two possible ways in which our method might produce less relevant or meaningless BPMs and discuss how to correct for each.

**Figure 4:**
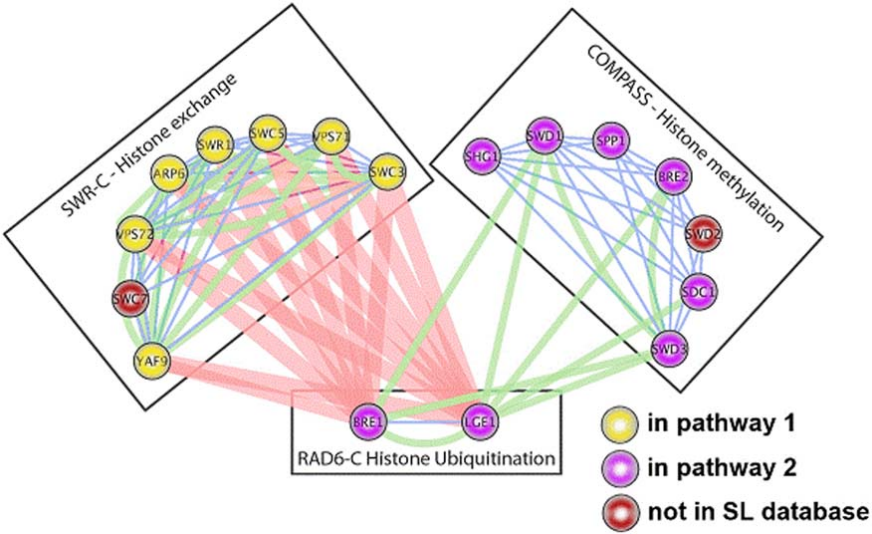
Tripartite pathway redundancy. This is a modified reproduction of structure C in Figure 5 in [34]. This structure is tripartite, with three interacting complexes. Our BPM 541 contains all but two of the genes involved in all three complexes (the two that are missing were not present in our synthetic-lethality data to begin with). BPM 541 correctly separates the complex on the left (yellow nodes are in pathway 1 of BPM 541) from the other two (violet nodes are in pathway 2 of BPM 541), but because our search is limited to bipartite structure, our algorithm grouped both the complex on the bottom and the one on the right together into a single “pathway,” basing this decision on the fact that there are more SL interactions observed between the bottom complex and the one on the left than were observed between the bottom complex and the one on the right.

First, there is the possibility of “fused pathways.” Our method only searches for *bipartite* structures, if there is a *tripartite* or multipartite redundancy arrangement, we may erroneously aggregate multiple pathways together into a single partition. We believe we have found at least one instance where this is happening (Figure 4).

A second potential issue is that when there are hub nodes (nodes of very high degree in the SL network), the structure of our algorithm will tend to give a high score to partitions that place the hub node in one partition and all of its neighbors in the other. In order to screen out these high-degree effects, on our website, we report results for alternative networks *G_75_*, *G_35_*, *G′_75_* and *G′_35_*, where for example *G_75_* stands for the subnetwork of *G* which remains after all genes of SL degree ≥75 have been deleted. Some interesting BPMs that are missed in the full network are uncovered in this way; we believe that more analysis of this effect is warranted in later studies.

### Future work

The present work makes use of only one class of genetic interaction, namely synthetic-lethality. There are other known classes of genetic interactions such as synthetic-sick and synthetic-rescue (when deletion of gene *A* has a particular phenotype distinct from wildtype, such as slow growth, but deletion of both *A* and *B* together results in a strain indistinguishable from wildtype). Supplementary results (reported on our website) imply that treating synthetic-sick interactions as equivalent to synthetic-lethality interactions (as Ulitsky and Shamir do) produces weaker results when using our method than limiting analysis only to the latter. We observed here that edges representing synthetic-lethality interactions behave as 2-vertex-cuts; it is not clear how best to incorporate other types of epistatic genetic interactions into our model. To extend this work to aid in the reconstruction of complete functional pathways – and not just fault-tolerant sub-mechanisms – we will also have to find ways to use evidence from purely physical interactions, so that all genes involved in each pathway can be placed back into pathways reconstructed solely from genetic interactions.

## Methods

### Data

We downloaded the genetic and protein-protein gene interaction networks used by Kelley and Ideker from their website [29]. We refer to this network as *G*. Our newer network *G′* was constructed from the BioGRID release 2.0.33 of Oct. 1, 2007. The SL network used to construct *G′* consisted simply of all SL interactions recorded for *S. cerevisiae*, along with all genes which participated in such interactions. The physical protein-protein interaction network used to validate BPMs from both genetic networks was also taken from this BioGRID release, and consisted of all interactions labeled as “Affinity Capture,” “Affinity Chromatography,” “Affinity Precipitation,” “Chip On-Chip,” “Co-Crystal Structure,” “Co-Purification,” “Phosphorylation Array,” “Purified Complex,” “Two-Hybrid,” “Protein-RNA,” “Protein-Peptide,” or “Reconstituted Complex.” Essential genes were filtered out before any processing took place; we retrieved a list of these genes from Stanford's “*Saccharomyces* Genome Deletion Project” website [30].

### Algorithm

We define the yeast SL graph *G* to have a vertex (node) corresponding to each gene/protein-product pair known to participate in at least one synthetic-lethality interaction, and an edge representing each such interaction. Let *G* have *n* vertices and *E* edges.

Given any bipartition (*A*,*B*) of *G* (that is, given any division of the nodes of *G* into two disjoint subsets *A* and *B*), let *c* denote the number of edges with one endpoint in *A* and one in *B*. For any vertex *v*∈*A*, define two new sets *A′* and *B′* to be *A*−{*v*} and *B*∪{*v*}, respectively. (Similarly, for *v*∈*B*, define *B′* to be *B*−{*v*} and *A′* to be *A*∪{*v*}.) We say that the bipartition (*A*,*B*) is *maximal in G* if the number of edges of *G* with one endpoint in *A′* and one in *B′* is at most *c*: in other words, moving a single vertex from *A* to *B* or vice versa cannot *increase* the number of edges that cross the cut between *A* and *B*.

In any partition (*A*,*B*) of the vertices of *G*, call a vertex *happy* if it has at least as many edges to vertices in the other partition as it does to vertices in its own partition, and *unhappy* otherwise. (The term “happy partition” was first used in [31].) The following procedure Flip generates a maximal bipartition of *G*; it is based on a classical result of Lovász [13].

1. Place each vertex of *G* into *A* or *B* uniformly at random.
2. While there exists at least one unhappy vertex in *G*:
  - Choose a random unhappy vertex *v*.
  - Switch its side (from *A* to *B* or from *B* to *A*).
3. Output the resulting sets *A* and *B*.

#### Theorem

Procedure Flip goes through its while loop at most *E* times, and results in a maximal bipartition of *G*.

#### Proof

Call an edge *crossing* if it has one endpoint in *A* and one endpoint in *B*. Each pass through the loop takes an unhappy vertex and makes it happy. This flip can have the side effect of causing previously happy vertices, which are neighbors of the flipped vertex, to become unhappy, leading to any given vertex potentially becoming happy and unhappy multiple times throughout the course of the algorithm. Every time the while loop is executed, however, *the number of crossing edges increases by at least one*, and there are *E* edges, so the loop terminates in at most *E* iterations. At termination, all vertices must be happy. Therefore, for each node, at least as many of its edges cross the partition as stay within a side of the partition. Thus, globally, there are at least as many edges that cross the partition as stay within a side of the partition. QED.

Running Flip several times may generate different maximal bipartitions, because of the random choices in initializing vertices to partitions, and also because of the random choices of which unhappy node to switch to happy at each iteration of the while loop. Notice that if we have a true example of the two-redundant-pathway BPM motif, there will be a large bipartite or nearly-bipartite subgraph contained in *G* whose SL edges are likely to cross the partition in “most” of the maximal bipartitions of *G* (because we get a large crossing gain for having the correct edges cross the partition). On the other hand, genes outside a BPM motif, who have close to a balanced number of SL edges to both pathways of a BPM motif, may appear as often on the *A* side as on the *B* side. So it seems desirable to prune out from a candidate BPM motif genes which *frequently switch sides* across different runs of the Flip algorithm: this motivates the following definition.

**Definition 1** *Given a gene v in G, run* Flip *M times on G. Label each gene with the number of times it appears on the* **same** *side as v in one of the M maximal bipartitions generated in this way, as well as with the number of times it appears on the* **opposite** *side from v. If gene w appears consistently (at least C% of the time) in the same partition as v, or consistently in the opposite partition from v, then w is included in the* **stable (bipartite) subgraph of** ***v***; *otherwise w is not included. The stable bipartite subgraph of v in G, then, is the subgraph induced by all included vertices, where v along with the vertices appearing consistently on the same side as v form one partition, and the rest of the included vertices form the other.*

Here *M* and *C*, the repetition threshold and the consistency threshold, respectively, are settable parameters of our method. All experiments in this paper used values of 250 and 70%, respectively, because these values of *M* and *C* generated the same or very similar stable bipartite subgraphs for each node *v*, across different runs of the randomized algorithm (see [32] for discussion of how these parameters were discovered and tuned). Thus we refer to “the” stable bipartite subgraph for *v* produced by this algorithm rather than “a” stable bipartite subgraph of *v*.

### GO enrichment calculations

Each BPM consisted of two sets of nodes (genes), representing two putative functional pathways exhibiting a redundant-backup relationship. We ran each pathway through FuncAssociate [16] (run with all default values, including for multiple testing correction, except with the significance threshold lowered from .05 to .01) to determine whether or not it was enriched for one or more GO terms. We also used the GOstat program [33] to calculate enrichment (using an FDR multiple testing correction) and results were quite similar; we report only FuncAssociate enrichment values because they are slightly more statistically conservative.

When running FuncAssociate on a particular pathway, we used the set of nodes in that pathway's source network (i.e., the BioGRID network or the Kelley-Ideker network) as a *background set* against which enrichment calculations were to be made, as a control against sampling biases in the networks themselves. GO enrichment was only counted for terms of depth at least 3 in the GO hierarchy (because enrichment for “biological_process,” for example – a top-level GO annotation term – is essentially meaningless for our purposes). We set the maximal *p*-value of the enrichment output to be 0.01; FuncAssociate uses a conservative familywise algorithm to correct against multiple testing errors.

### *p*-values for physical and new-SL edge distributions within BPMs

Given a BPM *X* = (*A*,*B*), constructed solely from SL edges, we wanted to overlay known PIs on top of *X* (say there were 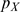 such interactions, with 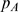 PIs between nodes in *A*, 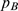 PIs between nodes in *B*, and 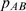 PIs with one endpoint in *A* and the other in *B*), and determine the probability that the observed bias of these PIs to appear *within* rather than *between* gene-sets *A* and *B* was due to random chance. To do this, we computed the probability, given a graph *G* with node set 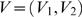 where 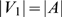, 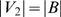 and 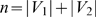, that we would see *at most* 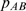 edges crossing *between* sets 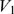 and 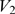, given an edge set *E* where 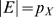, where edges in *E* were placed independently and uniformly at random between pairs of nodes. The formula for this is
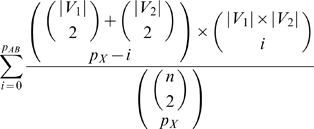



Similarly but conversely, given a BPM *X* = (*A*,*B*) derived from *G* (the original Kelley-Ideker network) and a set of 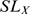 new SL edges appearing between pairs of nodes in *X*, to determine the probability that the observed bias of these new SL edges to appear *between A* and *B* rather than remaining *within* either *A* or *B* was due to random chance, the formula becomes
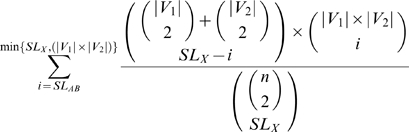



### Clustering: BPM redundancy

We used a straightforward clustering scheme to marginally reduce redundancy in the BPMs generated by our method: specifically, if two genes generated *identical* stable bipartite subgraphs, we merged the two and reported them as a single BPM. We note that this extremely conservative clustering method certainly results in some redundancy in reporting; there was no obviously justifiable place to set an “overlap threshold” (i.e., BPMs which overlap by ≥*X*%, or by ≥*X* genes, are considered “the same BPM,” while BPMs overlapping by <*X*% are “different BPMs”). For example, on the BioGRID network, when the overlap threshold was lowered in increments of 10% from 100% down to 10%, the number of clusters (putatively unique BPMs) generated at each threshold level were: 1,510 (100% – the threshold we report – which contains some redundancy but definitely does not merge unrelated BPMs), 867 (90%), 555 (80%), 299 (70%), 163 (60%), 84 (50%), 65 (40%), 56 (30%), 53 (20%) and 52 (10%). In the absence of a clear cutoff point, we decided only to cluster two stable subgraphs into a single BPM if they were exactly identical. We note further, however, that the number of SL edges covered by our total BPM set, as reported in Table 1, contains no redundancy: 4,949 *distinct* SL interactions were covered by our BPMs.
